# Impact of COVID-19 Mitigation Policy in South Korea on the Reduction of Preterm or Low Birth Weight Birth Rate: A Single Center Experience

**DOI:** 10.3390/children8050332

**Published:** 2021-04-25

**Authors:** Sae-Yun Kim, So-Young Kim, Kicheol Kil, Young Lee

**Affiliations:** 1 Department of Pediatrics, Yeouido St. Mary’s Hospital, College of Medicine, The Catholic University of Korea, Seoul 06591, Korea; sysmile@gmail.com; 2 Department of Pediatrics, Eunpyeong St. Mary’s Hospital, College of Medicine, The Catholic University of Korea, Seoul 06591, Korea; 3 Department of Obstetrics and Gynecology, Yeouido St. Mary’s Hospital, College of Medicine, The Catholic University of Korea, Seoul 06591, Korea; kilssine@catholic.ac.kr (K.K.); ly@catholic.ac.kr (Y.L.)

**Keywords:** preterm birth, low birth weight, social distancing, COVID-19

## Abstract

The government of South Korea implemented social distancing measures to control the coronavirus disease 2019 (COVID-19) outbreak. This study aimed to compare the composite preterm (PT) or low birth weight (LBW) birth rates during the COVID-19 pandemic period in South Korea to those during the prior decade, and to find out the associations of childbirth during the pandemic period with PT or LBW births. Over a ten-year period, this retrospective cohort study was performed in a single hospital in the Seoul metropolitan city. The COVID-19 period was defined as running from 22 March 2020, to 31 October 2020, and the pre-COVID-19 period as the sum of parallel periods from 2011 to 2019. Trends in composite birth rates were investigated, and logistic regression analysis was conducted to investigate independent factors associated with composite births. There were 246 and 2765 singleton deliveries during the COVID-19 period and the pre-COVID-19 period, respectively. The composite birth rate decreased from 16.5% to 9.8%. Childbirth during the pandemic was independently associated with a decreased composite birth rate (adjusted odds ratio, 0.563; 95% confidence interval, 0.355−0.844, *p* = 0.015). These findings suggested that the COVID-19 pandemic might provide an opportunity to find out preventive factors for PT or LBW births.

## 1. Introduction

Preterm (PT) birth is defined as all births before 37 weeks of gestation [1], the PT birth rate is approximately 10% worldwide [2], and was 8.1% in South Korea in 2019 according to a press release of the Korea National Statistical Office. Prematurity is associated with an increased risk of mortality and long-term morbidities and is the dominant cause of death in infants younger than five years of age, globally [2]. Birth weight is also a strong indicator evaluating the risk of perinatal mortality and morbidity because it is positively associated with gestational age (GA) [3]. Low birth weight (LBW) may result from PT birth or intrauterine growth restriction. LBW infants are exposed to an increased risk of several health problems such as growth retardation, infectious diseases, and developmental delay, similar to PT infants. Therefore, both PT and LBW infants could contribute to major public healthcare problems. The etiology of a PT or LBW infant birth is not yet fully understood because it is related to multiple sociodemographic, nutritional, biological, and environmental factors [2].

The World Health Organization declared the novel coronavirus disease 2019 (COVID-19) outbreak a global pandemic on 11 March 2020, as the number of confirmed cases outside China increased 13-fold and the number of countries with cases multiplied 3-fold [4]. After this declaration, many countries who did not begin to take action were under coercion to execute social distancing or lockdown as methods to control the transmission of the severe acute respiratory syndrome coronavirus 2 (SARS-CoV-2), the causative strain of COVID-19. Unlike many western countries, the government of South Korea chose to implement social distancing measures rather than a nationwide lockdown. Quarantine due to COVID-19 drastically modified daily lives by changing living conditions at home and working environments, reducing physical activities, and increasing the focus on hygiene. This uncommon situation is likely to have influenced several risk factors for PT or LBW birth. 

Recent studies on the effects of COVID-19 quarantine on PT or LBW infant birth have shown inconsistent results. High rates of PT birth have been reported in women with SARS-CoV-2 infection in a systematic review and meta-analysis conducted in the UK [5]. Moreover, a retrospective cohort in the UK and a prospective observational study in Nepal have also demonstrated a high PT birth rate during the COVID-19-related lockdown period [6,7]. In contrast, a Danish retrospective cohort study reported lower rates of PT birth at <28 weeks of gestation during the COVID-19 pandemic [8], and a nationwide study from the Netherlands [9] reported reductions in PT birth during quarantine. In terms of birth weight, the births of very low birth weight (VLBW) and extremely low birth weight (ELBW) infants unprecedentedly reduced during the COVID-19 pandemic period in Ireland [10]. These studies included confirmed cases only [5], analyzed relatively short study periods [6], made comparisons with unparallel periods [6,7], and used a single criterion of birth weight or GA [8,10]. Therefore, in the present study, which focused on composite PT or LBW birth rate, we aimed to find out the change in PT or LBW birth rate and maternal and neonatal characteristics during the COVID-19 pandemic period compared to data from the prior decade. Additionally, we investigated the associations of childbirth during the COVID-19 pandemic period and PT or LBW births.

## 2. Materials and Methods

### 2.1. Study Design and Population

The first imported case of COVID-19 was confirmed in South Korea on 20 January 2020 [11]. As the number of confirmed cases expanded exponentially, the South Korean population raised the level of alertness and the government began to take strict social distancing measures as opposed to enforcing a lockdown like many Western countries. Social distancing was enforced by the South Korean government on 22 March 2020; therefore, in this study, the period between 22 March 2020, and 31 October 2020, was denoted as the “COVID-19 period.” The “pre-COVID-19 period” was denoted as the serial sum of nine parallel periods from 2011 to 2019, when people were going about their normal daily lives without limitations. In South Korea, the confirmed cases were hospitalized in government-designated isolation hospitals; this also applied to pregnant women who had to visit hospital for prenatal care or delivery. 

This is a retrospective cohort study of pairs of mothers and their newborn babies; mothers who delivered at the Catholic University of Korea, Yeouido St. Mary’s Hospital from 2011 to 2020. Yeouido St. Mary’s Hospital is a secondary level general hospital based in an urban setting, located in Seoul metropolitan city area. There are approximately 500 deliveries annually, and about 400 high-risk infants who are admitted in NICU per year; among them, about 200 of infants born in our hospital are admitted to the NICU. All singleton pregnant women who delivered at ≥20 weeks of gestation during the study period in Yeouido St. Mary’s Hospital were included with their newborns. To limit the influence of other determinants of premature birth or birth weight, we excluded women with multiple pregnancies and their twin babies in the current study. This is not a designated hospital so hospitalization of women who were confirmed COVID-19 infection was restricted. This study was approved by the Institutional Review Board of Yeouido St. Mary’s Hospital, and informed consent was waived.

First, we examined the annual trends of the maternal characteristics and neonatal outcomes of singleton pregnancies at the Yeouido St. Mary’s Hospital in South Korea over a 10-year period, and then compared the differences in maternal and neonatal outcomes between the COVID-19 period and pre-COVID-19 period. Lastly, we identified independent factors that were associated with the birth of PT or LBW infants.

### 2.2. Definitions

An elderly primigravida is defined as a woman who becomes pregnant at an age of ≥35 years [12]. Maternal weight gain (kg) was calculated by subtracting the predelivery weight from the prepregnancy weight. Hypertensive disorders of pregnancy (HDP) were defined by the National High Blood Pressure Education Program Working Group on High Blood Pressure in Pregnancy [13]. Diabetes mellitus (DM) included both gestational DM diagnosed during the current pregnancy and overt DM. Intrapartum fever was defined as a rise in maternal body temperature above normal (>37.5 °C) during labor. Premature rupture of membrane (PROM) was defined as the rupture of fetal membranes before the onset of labor [14]. Stillbirth was defined as fetal death after 20 weeks of pregnancy [15]. PT birth was defined as the birth of a baby at less than 37 weeks of gestation, and very preterm (VP) and extremely preterm (EP) births were defined as births of babies less than 32 and 28 weeks of GA, respectively. LBW, VLBW and ELBW were defined as birth weights less than 2500 g, 1500 g and 1000 g, respectively. 

Maternal demographic data collected included age and weight. Antepartum factors included parity and maternal comorbidities such as hypertension and diabetes. The intrapartum factors assessed included PROM, mode of delivery, intrapartum fever, predelivery hemoglobin. The neonatal characteristics assessed included GA at birth, birth weight, gender, Apgar score (AS), incidence of NICU admission, and length of admission and mortality. 

### 2.3. Statistical Analysis

Statistical analysis was performed using SPSS version 25 (IBM Corp, Armonk, New York, USA). Ten-year trends were analyzed using the Mantel–Haenszel test of trend (linear-by-linear association) or one-way ANOVA test; continuous variables are presented as means and standard deviations, and dichotomous variables are presented as frequencies. Unadjusted comparisons of maternal and obstetrical characteristics and neonatal outcomes between the COVID-19 period and the pre-COVID-19 period were performed using Pearson’s chi-square or Fisher’s exact test for categorical data and Student’s t-test or the Mann–Whitney U test for continuous data. Factors found to be significant in the univariate analyses were included in the multivariate binary logistic regression model to identify associations with PT or LBW birth. All tests were two-tailed, and *p*-values < 0.05 were considered statistically significant.

## 3. Results

### 3.1. Ten-Year Trend of the Study Participants

Over the 10-year period, 5269 pregnant women delivered 4905 single children and 364 pairs of twins in the study hospital. Among them, we included 2765 singleton pregnant women and their infants born from 22 March to 31 October. There were changes in several factors. Maternal age at delivery increased, and the differences in annual mean maternal age at delivery were statistically significant (*p* < 0.001). After post hoc analysis, the differences between 2011 and the COVID-19 period, between 2012 and the COVID-19 period, and between 2011 and the other nine years were statistically significant. Overall, during the 10-year period, the number of women who became pregnant for the first time at the age of 35 years or more increased significantly (*p* < 0.001). The number of deliveries by cesarean section also increased (*p* < 0.001), and the hemoglobin level before delivery showed an increasing tendency over the 10-year period (*p* = 0.004). There were no significant differences in maternal weight gain during pregnancy throughout the 10-year period. Obstetric characteristics such as HDP, DM, PROM, and intrapartum fever showed no significant differences over the 10-year period. The PT, VP, and EP birth rates decreased but only a decreasing trend of VP birth rate was statistically significant (*p* = 0.043). The mean birth weight was not significantly different; however, the LBW, VLBW, ELBW, and the PT or LBW birth rate reduced, the trend of change over 10 years was not statistically significant; however, the number of infants admitted to the NICU increased significantly over the 10-year period (*p* < 0.001) (Table 1). 

### 3.2. Characteristics of the COVID-19 Period

Elderly primigravida women significantly increased from 11.7% (324/2765) to 22.4% (55/246) between the pre-COVID-19 period and the COVID-19 period (*p* < 0.001). Maternal weight gain during pregnancy and obstetrical characteristics such as HDP, DM, PROM, and intrapartum fever were not significantly different between the COVID-19 and pre-COVID-19 periods.

The mean GA at birth of infants during the COVID-19 period was 38 ^6/7^ ± 2 ^0/7^ weeks and that during the pre-COVID-19 period was 38 ^4/7^ ± 2 ^6/7^ weeks; the former was significantly older than the latter. The PT birth rate in the COVID-19 period was 8.5% (21/246), which was significantly lower than that in the pre-COVID-19 period, 14.5% (401/2765) (95% confidence interval (CI, 0.347–0.871, *p* = 0.010). Both the VP and EP birth rates also decreased during the pre-COVID-19 period (3.9% (108/2765) and 1.8% (49/2765), respectively) and during the COVID-19 period (1.2% (3/246) and 0.8% (2/246), respectively); only the VP birth rate showed a significant difference (*p* = 0.032) (Table 2). The COVID-19 period (2020) was set as the reference year to confirm that this was a coherent finding compared with previous year, and the adjusted odds ratio (aOR) of the PT, LBW, and VLBW birth rates and the PT or LBW birth rate were higher in all of the preceding years. In particular, the aOR of PT or LBW birth rate was 1.537–2.317 times higher in every antecedent years, although only five of the nine years showed statistical significance (Table S1).

The mean birth weight of infants born in the COVID-19 period was heavier than that of infants born in the pre-COVID-19 period (3190.7 ± 507.3 g and 3097.3 ± 654.6 g, respectively, *p* = 0.007). There were significant differences in LBW and VLBW birth rates; the LBW birth rate was 5.7% (14/246) and 12.5% (347/2765) in the COVID-19 period and pre-COVID-19 period, respectively (*p* = 0.002). The VLBW birth rate was 1.2% (3/246) and 3.6% (100/2765) in the COVID-19 period and pre-COVID-19 period, respectively (*p* = 0.032). We analyzed the composite birth rate from two perspectives, birth weight and gestation. The composite PT or LBW birth rate in the COVID-19 period was 9.8% (24/246), which was significantly lower than the 16.5% (456/2765) in the pre-COVID-19 period (OR, 0.547; 95% CI, 0.355–0.844, *p* = 0.006). The proportion of infants admitted to the NICU was significantly increased from 26.7% (737/2765) in the pre-COVID-19 period to 51.2% (126/246) in the COVID-19 period (*p* < 0.001) (Table 2).

During the COVID-19 period, more mature infants in terms of gestational age, more heavier infants who were admitted to the NICU were born, compared to the pre-COVID-19 period. Infants whose 5-min AS was < 7 accounted for 4.0% (5/126) in the COVID-19 period and 13.3% (98/737) in the pre-COVID-19 period (*p* = 0.003). The length of admission of high-risk infants born during the COVID-19 period was significantly longer than those during the pre-COVID-19 period (10.7 ± 11.1 days and 20.0 ± 22.6 days, respectively, *p* < 0.001) (Table S2).

### 3.3. PT or LBW Birth Rates

Multivariate logistic regression analysis was performed to adjust for all possible confounding factors associated with PT or LBW birth rate in the study participants during the two study periods. The following parameters were assessed in the multivariate model: maternal age, maternal weight gain, HDP, PROM, hemoglobin level before delivery, and childbirth during the COVID-19 period. Maternal age at delivery (aOR, 0.948; 95% CI, 0.923–0.974; *p* < 0.001), maternal weight gain during pregnancy (aOR, 0.854; 95% CI, 0.831–0.877; *p* <0.001), HDP (aOR, 7.523; 95% CI, 5.154–10.980; *p* < 0.001), PROM > 24 h (aOR, 6.025; 95% CI, 4.462–8.134; *p* < 0.001), and childbirth during the COVID-19 period (aOR, 0.563; 95% CI, 0.355–0.893; *p* = 0.015) were independently associated with the birth of PT or LBW infants (Table 3). 

## 4. Discussion

Several remarkable findings were noted in the current study, which analyzed data from more than six months over the COVID-19 pandemic. First, we demonstrated that the composite PT or LBW birth rate during the COVID-19 period was lower than that during the pre-COVID-19 period: 9.8% (24/246) vs. 16.5% (456/2765). Second, we documented distinct evidence of the independent association between childbirth during the COVID-19 period and PT or LBW birth, while considering maternal age at birth, maternal weight gain during pregnancy, HDP, PROM, and hematologic factors before delivery as confounding factors. Third, although more infants were admitted to the NICU in the COVID-19 period, the severity estimated by GA at birth, birth weight, AS, and length of admission were milder in the COVID-19 period than in the pre-COVID-19 period, which resulted in improved neonatal outcomes.

According to our results, maternal age, weight gain, HDP, PROM, and childbirth during the COVID-19 pandemic were independently associated with PT or LBW birth. Maternal old age, HDP, and PROM were common causes of PT birth. Weight gain during pregnancy was usually proportional to gestational age. Meanwhile, childbirth during the COVID-19 pandemic lowered the occurrence of PT or LBW birth by a factor of 0.56, which is an interesting finding. The birth of PT or LBW infants is a significant medical, psychological, emotional, and financial burden for affected infants, their families, health systems, and society in general and may result in significant neonatal morbidities, leading to long-term health concerns. Features of high-risk pregnancy were found in our study. With older maternal ages, the proportion of elderly primigravida and mothers with diabetes increased during the COVID-19 period. These changes could be associated with the current global trend of high-risk pregnancies and PT birth increase. However, discordant with the recent increase in PT birth rate with decreasing total birth rate in South Korea [16], we found out that the observed birth rate reduced in all preterm groups during the COVID-19 period, although the changes in trend showed statistical significance only in the VP group. The complicated socioeconomic crisis due to the restrictions to daily life for the control of disease transmission may have led to the reduction of total and PT births.

As stated in a recent systematic review and meta-analysis of SARS-CoV-2 infection and pregnancy, PT birth was common (21.8%) in infected pregnant women [5], indicating that the risk of iatrogenic PT birth was also increased. In contrast, pregnancy complications, including PT or LBW birth, decreased in the general population during the pandemic era. The current reduction of PT or LBW birth rate found in this study is the result of the complex various socioenvironmental changes. The changes during the COVID-19 period have also affected through pandemic-related issues, pregnancy and neonatal outcome. The exact mechanism and reasons have not yet been identified, and paradoxical health improvement might be one possible explanation. Exposure to air pollution during pregnancy has been associated with the risk of PT birth [17]. Inhalation of polluted air increases toxic chemicals in the blood and stresses the immune system. It may also be associated with an unfavorable vaginal microbiota. In South Korea, with an emphasis on social distancing, human activity restrictions, such as intercountry movement and reduced plant operations, maximized improvements in air quality due to reduced air pollutants [18]. These changes modified pregnancy, with a positive influence on health.

Common causes of PT birth include maternal old age, and maternal chronic conditions did increase during the COVID-19 period. Infection might be an important cause of PT birth. Another possible explanation for the lower PT and LBW birth rates include improved personal hygiene and reduced personal contact; this could allow mothers to avoid other dangerous infections that lead to maternal systemic inflammation and subsequent pregnancy complications. In our study, women with intrapartum fever increased during the COVID-19 period without statistical significance. This was because medical staff thoroughly screened every pregnant woman during the COVID-19 period and body temperature over 37.5 °C could not be interpreted as infection.

Another explanation cautiously suggested was that relative hypoxia resulting from a face mask could cause heme oxygenase-1 induction. Heme oxygenase-1 strengthens hemoglobin production and has been shown to reduce spontaneous PT birth rate [19]. We found that the hemoglobin level during the COVID-19 period was higher than that during the pre-COVID-19 period, but the mean value of hemoglobin throughout the 10 year period taken annually was not in line with every year’s PT birth rate.

While the prevalence of PT or LBW decreased in the COVID-19 period, the rate of admission to NICU has increased considerably. Operating NICUs in South Korea did not have economic efficiency as the government policies changed in the 2010s [20]. Hospitalization of high-risk infants in need of neonatal intensive care has become easier than in the past, making it possible for them to receive appropriate treatment in a timely manner, and infants with milder symptoms can be hospitalized as compared to the past. This finding was confirmed by our results; infants with older gestational age, heavier birth weight, and higher 5 minutes AS were admitted to the NICU during the COVID-19 period, and their length of hospital stay was shorter. As a result, the total number of hospitalized infants increased during theCOVID-19 period.

Several previous studies controlled or observed one or two variables; however, in the current study, multiple confounding factors that could affect the birth of PT or LBW infants were controlled by logistic regression analysis, as childbirth during the COVID-19 period still had an independent relationship with the PT or LBW birth along with maternal HDP and PROM. Paradoxically, the COVID-19 pandemic has been an opportunity for the general population to raise awareness of the importance of maternity health. Although not statistically significant, there was a concurrent decrease in stillbirth between the two periods in our study population, which is one of important pregnancy outcomes. It is too early to assess the exact cause-and-result relationship that led the unidentified reasons to positively contribute to pregnancy and neonatal outcomes. 

The main strength of the current study is that we are the first to find out the reduction of composite birth rate of PT and LBW infants. Second, we analyzed a relatively long pandemic period, more than six months and additionally third, we analyzed multiple obstetrical and neonatal characteristics during the pandemic period and compared them with the results over the past decade. Lastly, we considered multiple confounders in order to evaluate the independent association between the birth of PB or LBW infants and the COVID-19 period. However, this study has a few limitations. First, this is a single-center, relatively small-sized observational study. Therefore, the generalization of these results to a larger population could produce biased results; the researchers should have a prudent attitude toward the interpretation of our study results. Second, despite the independent association of the COVID-19 period and the birth of PT or LBW infants, a causal relationship between the two factors could not be established. Third, the increase in women’s social advancement is related to the fertility rate or birth rates. Although the decrease in the number of premature births observed in this study is independent of the pandemic era, it also included social factors, and it is impossible to evaluate both of these completely, yet separately. Lastly, we compared the parallel period to remove the seasonality effect; however, there might be seasonal variations between March and October.

## 5. Conclusions

The composite birth rate of PT or LBW infants in South Korea markedly decreased with social distancing measures during the COVID-19 pandemic. The exact mechanism of these phenomenon was not completely known; however decreased air pollution, involvement with personal hygiene, and maternal infection risk might be possible causes. Considering that PT birth is one of the most important causes of neonatal death world-wide and the survivors are at greater risk of short-term morbidities, impairment and long-term neurodevelopmental impairments, there is a strong need to find out the hidden reasons for the unpredicted reduction in PT or LBW birth rate revealed during the COVID-19 pandemic period and improve children’s public health. Further research is required to better understand the pathogenesis underlying the lower PT and LBW birth rate infants during the pandemic period.

## Figures and Tables

**Table 1:** Ten years trend of maternal and neonatal characteristics.

	2011	2012	2013	2014	2015	2016	2017	2018	2019	2020	*p*
**No. of deliveries (single/total)**	344/370	337/375	267/301	247/279	225/257	287/361	329/379	368/436	361/413	246/268	-
**Maternal age, years**	32.3 ± 4.1	33.1 ± 4.2	33.2 ± 4.0	33.2 ± 3.9	33.5 ± 3.8	33.7 ± 4.0	33.4 ± 4.0	33.4 ± 4.2	33.5 ± 4.1	34.3 ± 4.1	<0.001
**Elderly primigravida**	15 (4.4)	21 (6.2)	22 (8.2)	18 (7.3)	24 (10.7)	49 (17.1)	54 (16.4)	57 (15.5)	64 (17.7)	55 (22.4)	<0.001
**Weight gain, Kg**	13.0 ± 4.9	12.8 ± 5.1	12.6 ± 4.9	13.2 ± 4.8	13.0 ± 5.1	13.0 ± 4.9	12.9 ± 4.8	13.7 ± 5.3	13.0 ± 4.9	12.9 ± 4.7	0.381
**HDP**	20 (5.8)	24 (7.1)	11 (4.1)	14 (5.7)	14 (6.3)	21 (7.4)	20 (6.1)	20 (5.4)	19 (5.3)	15 (6.1)	0.833
**DM**	18 (5.2)	17 (5.0)	17 (6.4)	23 (9.3)	19 (8.4)	18 (6.3)	17 (5.2)	29 (7.9)	26 (7.2)	24 (9.8)	0.064
**Intrapartum fever**	11 (3.2)	28 (8.3)	9 (3.4)	6 (2.4)	1 (0.4)	5 (1.7)	17 (5.2)	24 (6.5)	18 (5.0)	5 (2.0)	0.802
**PROM > 24 h**	29 (8.4)	32 (9.5)	26 (9.7)	21 (8.5)	15 (6.7)	21 (7.3)	31 (9.4)	27 (7.3)	30 (8.3)	23 (9.3)	0.693
**C/S**	93 (27.0)	78 (23.1)	68 (25.5)	86 (34.8)	80 (35.6)	108 (37.6)	122 (37.1)	143 (38.9)	128 (35.5)	97 (39.4)	<0.001
**Hb before delivery, g/dL**	11.78 ± 1.34	11.88 ± 1.36	11.92 ± 1.39	11.91 ± 1.40	11.83 ± 1.38	12.11 ± 1.42	12.16 ± 1.29	11.98 ± 1.32	12.01 ± 1.29	12.11 ± 1.28	0.004
**Male infant**	160 (46.5)	159 (47.2)	140 (52.4)	123 (49.8)	109 (48.4)	136 (47.4)	155 (47.1)	201 (54.6)	189 (52.4)	122 (49.6)	0.125
**GA at birth, week**	38 ^3/7^ ± 3 ^2/7^	38 ^4/7^± 3 ^0/7^	38 ^5/7^ ± 2 ^6/7^	39 ^0/7^ ± 2 ^0/7^	38 ^3/7^ ± 3 ^0/7^	38 ^5/7^ ± 3 ^0/7^	38 ^3/7^ ± 3 ^3/7^	38 ^4/7^ ± 2 ^3/7^	38 ^4/7^ ± 2 ^6/7^	38 ^6/7^ ± 2 ^0/7^	0.115
**<37 ^0/7^**	60 (17.4)	47 (13.9)	34 (12.7)	29 (11.7)	32 (14.2)	37 (12.9)	53 (16.1)	58 (15.8)	51 (14.1)	21 (8.5)	0.211
**<32 ^0/7^**	15 (4.4)	17 (5.0)	12 (4.5)	4 (1.6)	10 (4.4)	12 (4.2)	17 (5.2)	10 (2.7)	11 (3.0)	3 (1.2)	0.043
**<28 ^0/7^**	9 (2.6)	6 (1.8)	4 (1.5)	0 (0.0)	6 (2.7)	6 (2.1)	10 (3.0)	2 (0.5)	6 (1.7)	2 (0.8)	0.282
**BW, g**	3074.8 ± 722.5	3136.2 ± 682.5	3138.7 ± 636.0	3149.5 ± 550.7	3021.5 ± 675.6	3107.8 ± 669.2	3036.5 ± 684.6	3117.1 ± 590.3	3089.9 ± 648.5	3190.7 ± 507.3	0.075
**LBW**	49 (14.2)	44 (12.2)	30 (11.2)	26 (10.5)	31 (13.8)	35 (12.2)	45 (13.7)	43 (11.7)	44 (12.2)	14 (5.7)	0.055
**VLBW**	15 (4.4)	14 (4.2)	7 (2.6)	4 (1.6)	12 (5.3)	12 (4.2)	15 (4.6)	10 (2.7)	11 (3.0)	3 (1.2)	0.149
**ELBW**	11 (3.2)	10 (3.0)	4 (1.5)	0 (0.0)	5 (2.2)	6 (2.1)	8 (2.4)	3 (0.8)	7 (1.9)	2 (0.8)	0.054
**PT or LBW**	68 (19.8)	52 (15.4)	40 (15.0)	37 (15.0)	37 (16.4)	44 (15.3)	59 (17.9)	64 (17.4)	55 (15.2)	24 (9.8)	0.093
**Stillbirth**	6 (1.7)	2 (0.6)	6 (2.2)	1 (0.4)	2 (0.9)	5 (1.7)	5 (1.5)	1 (0.4)	4 (1.1)	1 (0.4)	0.209
**NICU admission**	78 (22.7)	83 (24.6)	55 (20.6)	63 (25.5)	57 (25.3)	71 (24.7)	105 (31.9)	121 (32.9)	106 (29.4)	126 (51.2)	<0.001

Included births each year took place between 22 March to 31 October. Values are means ± standard deviation or frequencies (percentage), as appropriate. *p* values calculated from Mantel−Haenszel test or one way-ANOVA from 2011 to 2020, as appropriate. BMI, body mass index; HDP, hypertensive disease of pregnancy; DM, diabetes mellitus; PROM, premature rupture of membrane; C/S, caesarean section; Hb, hemoglobin; GA, gestational age; BW, birth weight; LBW, low birth weight; VLBW, very low birth weight; ELBW, extremely low birth weight; PT or LBW, preterm or low birth weight; NICU, neonatal intensive care unit.

**Table 2:** Comparison of between COVID-19 period and pre-COVID-19 period.

	COVID-19 (*N* = 246)	Pre-COVID-19 (*N* = 2765)	OR (95% CI)	*p* Value
**Maternal age, years**	34.3 ± 4.1	33.3 ± 4.1	-	<0.001
**Elderly primigravida**	55 (22.4)	324 (11.7)	2.169 (1.573–2.992)	<0.001
**Weight gain, Kg**	12.9 ± 4.7	13.0 ± 5.0	-	0.717
**HDP**	15 (6.1)	163 (5.9)	1.033 (0.598–1.782)	0.908
**DM**	24 (9.8)	184 (6.7)	1.516 (0.970–2.371)	0.066
**Intrapartum fever**	5 (2.0)	119 (4.3)	0.461 (0.187–1.140)	0.086
**PROM > 24 h**	23 (9.3)	232 (8.4)	1.126 (0.718–1.766)	0.605
**C/S**	97 (39.4)	906 (32.8)	1.336 (1.022–1.746)	0.034
**Hb before delivery, g/dL**	12.11 ± 1.28	12.0 ± 1.4	-	0.098
**Male infant**	122 (49.6)	1372 (49.6)	0.999 (0.770–1.297)	0.994
**GA at birth, wk**	38 ^6/7^ ± 2 ^0/7^	38 ^4/7^ ± 2 ^6/7^	-	0.034
**<37 ^+0^**	21 (8.5)	401 (14.5)	0.550 (0.347–0.871)	0.010
**<32 ^+0^**	3 (1.2)	108 (3.9)	0.304 (0.096–0.964)	0.032
**<28 ^+0^**	2 (0.8)	49 (1.8)	0.454 (0.110–1.880)	0.434
**BW, g**	3190.7 ± 507.3	3097.3 ± 654.6	-	0.007
**LBW**	14 (5.7)	347 (12.5)	0.421 (0.242–0.730)	0.002
**VLBW**	3 (1.2)	100 (3.6)	0.329 (0.104–1.045)	0.047
**ELBW**	2 (0.8)	54 (2.0)	0.412 (0.100–1.698)	0.320
**PT or LBW infant**	24 (9.8)	456 (16.5)	0.547 (0.355–0.844)	0.006
**Stillbirth**	1 (0.4)	32 (1.2)	0.349 (0.047–2.562)	0.516
**NICU admission**	126 (51.2)	737 (26.7)	2.889 (2.219–3.762)	<0.001

Values are means ± standard deviation or frequencies (percentage), as appropriate. *p* values calculated from Student’s *t*-test or the Pearson Chi square test, as appropriate. HDP, hypertensive disease of pregnancy; DM, diabetes mellitus; PROM, premature rupture of membrane; C/S, Cesarean section; Hb, hemoglobin; GA, gestational age; BW, birth weight; LBW, low birth weight; VLBW, very low birth weight; ELBW, extremely low birth weight; PT or LBW, preterm or low birth weight; NICU, neonatal intensive care unit.

**Table 3:** Factors independently associated with PT or LBW birth.

	PT/LBW (*N* = 480)	FT/NW (*N* = 2531)	OR (95%CI)	*p*	aOR (95%CI)	*p* *
**Maternal age, years**	32.7 ± 4.6	33.5 ± 4.0	-	0.001	0.948 (0.923–0.974)	<0.001
**Elderly primigravida**	67 (14.0)	312 (12.3)	1.154 (0.868–1.533)	0.323	-	-
**Weight gain, Kg**	10.1 ± 5.0	13.6 ± 4.8	-	0.000	0.854 (0.831–0.877)	<0.001
**HDP**	69 (14.5)	109 (4.3)	3.758 (2.731–5.171)	0.000	7.523 (5.154–10.980)	<0.001
**DM**	32 (6.7)	176 (7.0)	0.956 (0.647–1.412)	0.820	-	-
**Intrapartum fever**	26 (5.4)	98 (3.9)	1.422 (0.912–2.216)	0.118	-	-
**PROM > 24 h**	112 (23.3)	143 (5.6)	5.082 (3.878–6.662)	0.000	6.025 (4.462–8.134)	<0.001
**Hb before delivery, g/dL**	11.8 ± 1.5	12.0 ± 1.3	-	0.047	0.927 (0.854–1.006)	0.068
**Childbirth during COVID-19 pandemic**	24 (5.0)	222 (8.8)	0.547 (0.355–0.844)	0.006	0.563 (0.355–0.893)	0.015

Values are means ± standard deviation or frequencies(percentage), as appropriate. *p* values calculated from Student’s *t*-test or the Pearson Chi square test. *p* * and aOR following logistic regression analysis include the following factors: maternal age, weight gain, HDP, PROM, Hb before delivery and childbirth during COVID-19 pandemic. PT/LBW, preterm or low birth weight; FT/NW, full term and normal birth weight (>2.5 kg); OR, odds ratio; aOR, adjusted odds ratio; HDP, hypertensive disease of pregnancy; DM, diabetes mellitus; PROM, premature rupture of membrane; Hb, hemoglobin.

## Data Availability

Not applicable.

## Supplementary Materials

The following are available online at https://www.mdpi.com/article/10.3390/children8050332/s1, Table S1: Odds of birth of PT or LBW infants in each year compared with year 2020, Table S2: Comparison of neonatal outcome between COVID-19 period and pre-COVID-19 period.
