# Supplementary material for: Impact of COVID-19 Mitigation Policy in South Korea on the Reduction of Preterm or Low Birth Weight Birth Rate: A Single Center Experience
Source: Children (Basel). 2021 Apr 25;8(5):332. doi: 10.3390/children8050332 (PMC8144961; doi:10.3390/children8050332)
Supplement: Supplementary file 1 [file children-08-00332-s001.zip › children-1169528 supplementary.pdf]

Table S1. Odds of birth of PT or LBW infants in each year compared with year 2020.

	2011	2012	2013	2014	2015	2016	2017	2018	2019
	aOR (95% CI)								
**GA, wks**									
**$< 37^{+0}$**	2.281 (1.290-4.035)*	1.591 (0.088-2.848)	1.410 (0.755-2.633)	1.348 (0.710-2.561)	1.833 (0.975-3.446)	1.482 (0.801-2.743)	1.993 (1.121-3.544)*	2.395 (1.121-3.544)*	1.878 (1.057-3.336)*
**$< 32^{+0}$**	3.280 (0.842-12.768)	3.996 (1.053-15.167)*	2.836 (0.694-11.599)	0.982 (0.191-5.055)	3.623 (0.886-14.814)	3.285 (0.817-13.213)	4.228 (1.110-16.109)*	2.432 (0.590-10.017)	2.793 (0.697-11.184)
**$< 28^{+0}$**	2.885 (0.519-16.030)	1.802 (0.305-10.636)	0.805 (0.112-5.786)		3.431 (0.582-20.229)	2.256 (0.372-13.685)	3.463 (0.637-18.832)	0.796 (0.094-6.745)	2.315 (0.388-13.797)
**LBW**	2.758 (1.422-5.351)*	2.377 (1.224-4.615)*	1.997 (0.984-4.052)	1.931 (0.938-3.977)	2.842 (1.400-5.769)*	2.280 (1.139-4.563)*	2.579 (1.329-5.013)*	2.590 (1.329-5.046)*	2.626 (1.355-5.090)*
**VLBW**	3.283 (0.864-12.470)	3.201 (0.850-12.063)	1.515 (0.342-6.712)	1.099 (0.221-5.464)	4.439 (1.138-17.314)*	3.035 (0.772-11.938_	3.667 (0.979-13.732)	2.384 (0.591-9.621)	2.757 (0.706-10.771)
**ELBW**	3.498 (0.674-18.153)	3.391 (0.659-17.450)	0.901 (0.130-6.254)		2.373 (0.404-13.931)	1.811 (0.311-10.5420)	2.610 (0.490-13.900)	1.049 (0.148-7.429)	2.562 (0.465-14.120)
**PT or LBW**	2.292 (1.339-3.924)*	1.548 (0.893-2.684)	1.537 (0.859-2.751)	1.618 (0.896-2.921)	1.908 (1.053-3.455)*	1.608 (0.904-2.858)	1.974 (1.146-3.4)*	2.317 (1.354-3.963)*	1.770 (1.027-3.051)*

*$p<0.05$. aOR; adjusted odds ratio for maternal age, maternal weight gain during pregnancy, hypertensive disorder of pregnancy, PROM. PT or LBW, preterm or low birth weight; aOR, adjusted odds ratio; LBW, low birth weight; VLBW, very low birth weight; ELBW, extremely low birth weight;.

Table S2. Comparison of neonatal outcome between COVID-19 period and pre-COVID-19 period.

	COVID-19 [N=246]	Pre-COVID-19 [N=2765]	OR (95% CI)	p value
**GA at birth**	$38^{4/7}$±$2^{2/7}$	$36^{4/7}$±$3^{6/7}$	-	<0.001
**BW, g**	3101.8±550.8	2671.5±865.8	-	<0.001
**5AS**	9.1±1.2	7.6±2.1	-	<0.001
**5AS<7**	5 (4.0)	98 (13.3)	0.270(0.108-0.678)	0.003
**Length of admission, d**	10.7±11.1	20.0±22.6	-	<0.001
**Mortality**	0 (0.0)	19 (2.6)	-	0.396

Values are means ± standard deviation or frequencies(percentage), as appropriate. *p* values calculated from Student's *t*-test or the Pearson Chi square test, as appropriate. GA, gestational age; BW, birth weight;
